# Turning Foetal Breech Presentation at 32-35 Weeks of Gestational Age by Acupuncture and Moxibustion

**DOI:** 10.1155/2019/8950924

**Published:** 2019-06-09

**Authors:** Paolo Brici, Giovanna Franconi, Cristina Scatassa, Elisabetta Fabbri, Paolo Assirelli

**Affiliations:** ^1^Emergency Unit, Ceccarini Hospital, Rimini Local Sanitary Unit, Riccione, Italy; ^2^Department of Systems Medicine, Tor Vergata University, Via Montpellier 1, 00133 Rome, Italy; ^3^ObGyn Unit, Consultorio Celle, Rimini Hospital and Rimini Local Sanitary Unit, Rimini, Italy; ^4^Research and Innovation, Rimini Hospital and Rimini Local Sanitary Unit, Rimini, Italy

## Abstract

**Introduction:**

Foetal breech presentation is an obstetric problem that often leads to caesarean section. Stimulation of the acupoint BL67 by moxibustion may correct breech presentation.

**Methods:**

We observed 93 pregnant women in the 32nd-35th week of gestation with normal pregnancy and ultrasound diagnosis of breech presentation. The patients received stimulation of acupoint BL67 by self-administered moxibustion once a day for two weeks and if foetuses still were in breech presentation, moxibustion, and needle in the points BL65 and SI1, lasting 30 minutes, for three days in one week. The main outcome was vaginal birth with vertex presentation at delivery; the secondary outcome was compliance in the self-administration of the moxibustion treatment.

**Results:**

We observed cephalic version and natural childbirth in 62.4% of all treated women. The treatment was accepted by 98.9% women (93/94), and compliance was 91.4% (85/93) for self-administered moxibustion and 37.5% (12/32) for moxibustion and needle treatment.

**Conclusions:**

On the basis of our results, self-administered home treatment moxibustion followed by moxibustion and needle stimulations may be an effective and low-cost treatment for inducing cephalic version.

## 1. Introduction

Vaginal delivery in breech presentation involves a morbidity rate of 4x compared to leaving for the cephalic end. Planned caesarean section reduces infant mortality and morbidity [1], but, according to the latest WHO estimates, it implies a morbidity rate of 2.7x compared to cephalic vaginal delivery [2]. Prior to 32 weeks of gestation, spontaneous version of the fetus to vertex position occurs more frequently than after 32 weeks [3]. At 32-35 weeks, 6-10% of foetuses are breech [4]. Spontaneous foetal reversal after the 32^nd^ week has not been well studied, but reported percentages range between 45% (no vertex at 35 weeks) [4] and 57% (no vertex at 32 weeks) [3].

It has been suggested that moxibustion, with or without acupuncture, practiced on the acupoint BL67 (Zhiyin) between the 32nd and 36th week may be effective for turning breech presentation. Since the 1970s, nonrandomized and randomized clinical trials have been conducted in China and in other countries on the use of moxibustion with or without acupuncture on BL67, the last point of the Bladder Meridian. Eight of these studies involving 1346 women have been reviewed in a Cochrane Collaboration systematic review [5], which concluded that moxibustion did not reduce the number noncephalic presentations at birth compared with no treatment but, when combined with acupuncture, moxibustion resulted in fewer noncephalic presentations at birth and fewer births by caesarean section. Given that the majority of trials were of moderate methodological quality but the interventions were very different, the conclusions from the meta-analyses should be interpreted with caution. A recommendation was made to include clinically relevant outcome measures, such as those relating to birth outcomes. A more recent systematic review [6] and a randomised trial [7] found a benefit in the use of “acupuncture-type interventions” on BL67, compared to expectant management or sham acupuncture, to induce the correction of breech presentation. Moxibustion is a procedure which is complementary and not alternative to the external turning manoeuvres [8], which are practiced in the 38th gestational week. External cephalic version is one of the most common procedures performed in the West to correct breech presentation with a manual procedure, with a success rate of between 35 and 57 per cent in nulliparous women [9]. However, this procedure presents some risk to both mother and baby and must be executed by a trained gynaecologist in a hospital, because in case of rupture of the membranes an emergency caesarean section may be required [10]. Moxibustion is considered safe both for the pregnant mother and for the foetus [11]. The New Zealand Guidelines Group for treatment for women with breech presentation recommend to offer moxibustion to women with breech presentation from the 33rd week [12].

**Figure 1:**
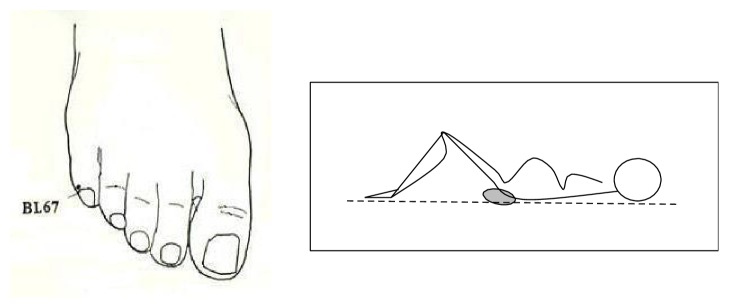
Drawings taken from the leaflet given to pregnant women for self-administration of moxibustion on BL67.

Most studies have examined the effect of moxibustion administered on pregnant women regardless of their position during the treatment. However in the ancient Chinese medical tradition from the oral Taoist teachings as explained by Master Jeffrey Chong Yuen it was recommended to treat the patient supine with knees and hips flexed, with a pillow placed under the sacral region for anterior tilt of the pelvis. In Chinese tradition, this position allows the “opening” of the Bladder Meridian (Figure 1). In case of failure of this treatment, we added another treatment with acupuncture and moxibustion on BL65 and SI1. According to Master Jeffrey Yuen, this is aimed at the umbilical cord, which the Chinese gynaecology school believed responsible for the noncephalic version despite treatment of BL67.

The purpose of the present study is to observe the effectiveness of moxibustion with or without needle acupuncture in women with breech presentation, when administered in the position to open the Bladder Meridian.

The main outcome of the study was vaginal birth with vertex presentation at delivery. Secondary outcomes were compliance in the self-administration of the moxibustion treatment and acceptance of needle-moxibustion treatment.

## 2. Methodology

Pregnant women were enrolled at the public gynaecology clinic “Consultorio Celle” of Rimini Local Sanitary Unit, in Rimini, Italy, from May 2008 to April 2010. The protocol was approved by the Ethics Committee of Area Vasta Romagna and IRST (Romagna Scientific Institute for the Study and Treatment of Cancer) with the Experimentation Registry number 91/07 in the session of November 21st, 2007. The protocol was not registered in any public registry.

Inclusion criteria were mother's age >18 years, uncomplicated pregnancy, ultrasound diagnosis of foetal breech at the 32nd to the 35th gestational week, and normal foetal biometry (biparietal diameter and abdominal circumference between 10° and 90° percentile). Exclusion criteria were participation in other experimental trials, twin pregnancies, pelvic defects, previous uterine surgery, uterine malformation or fibromyoma diameter greater than 4 cm, previous caesarean section, risk of premature birth (uterine hypercontractility and / or initial shortening or dilatation of the cervix with a Bishop score of ≥ 4), or pathological pregnancy (e.g., intrauterine growth retardation, gestosis, serious infections, placenta previa, polyhydramnios, and oligohydramnios).

If a woman met the inclusion criteria she was asked if she agreed to treatment with self- administered moxibustion and needle-moxibustion. In case of agreement the woman signed a written informed consent and the foetal position was rechecked by ultrasound if the previous one had been done more than 24 hours before. Women were then trained to self- administer moxibustion. They were instructed to lie supine with knees and hips flexed, with no external constraint on the abdomen and to place a pillow under the sacral region to tilt the pelvis (see Figure 1). Treatment consisted in heating the point BL67 bilaterally with moxibustion for 15-20 min daily. The heating was done by approaching the lit point of the Artemisia Vulgaris cigar to the skin and to turn it away temporarily if and when the sensation of heat became unpleasant. The partner was also trained in this task.

**Figure 2:**
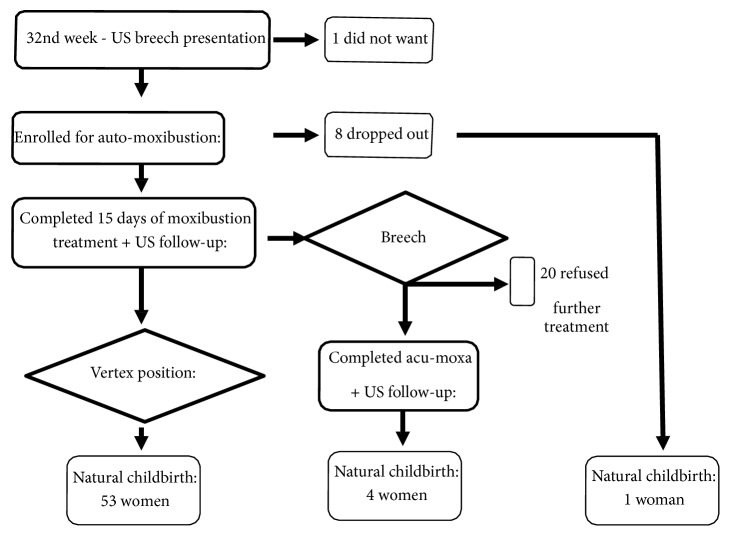
Flow chart of the study.

After two weeks of home self-treatment a control ultrasound was performed in all pregnant women to evaluate the foetal response. At the end of the second week, in case of the persistence of abnormal foetal presentation, a new treatment was started which consisted in acupuncture and moxibustion on BL65 and SI1 for the duration of 30 minutes, three times in one week. We used acupuncture needles 0.25 x 25mm (Huanqiu, Suzhou, PRC), with a very shallow insertion on SI1 and a 2 mm insertion at BL65. The acupuncture treatment was done by a single experienced acupuncturist (PB). A midwife (CS) collected the data and organized the next step. The outcome of vaginal birth with vertex presentation was investigated by contacting the treated women by telephone after delivery. A flow diagram elucidating the study program is shown in Figure 2.

The patients were evaluated according to the principle of “Intention to treat.” All qualitative variables were analysed in terms of frequency and the quantitative variables in terms of mean, median, and standard deviation. The statistical analysis of data included the use of Pearson chi square test for the relation between single variables and foetal reversals, and significance was reached if P <0.05.

## 3. Results

During the 2 years of enrolment, 94 pregnant women met the inclusion criteria and 93 agreed to participate in the study. The woman who did not enter the study did not sign the informed consent because the husband did not agree.

The sample was composed of 60 primiparae and 33 multiparae aged 18 to 45 years, mean age 32 years. Seventy-seven women were Italian citizens; the other 16 were foreign women living in Italy, distributed as follows: 7 coming from the Balkans, 4 from Eastern Europe, 3 from the Maghreb, 1 Iranian, 1 Chinese. Mean gestational week at the beginning of the treatment was 33.9 (range 33 to 36).

Of the 93 enrolled women, 85 (91.4%) successfully self-administered the moxibustion treatment. One enrolled woman stated she could not maintain the supine position and we recommended performing the treatment lying on the side. Among the women who did not succeed in self-administering the treatment, one woman did so because of intolerance to the moxibustion smoke and the others because of lack of interest or family difficulties. After 2 weeks of moxibustion treatment, 32 (33,7%) pregnant women did not present foetal reversal. Twelve women of them (12.9%) continued the treatment for 3 more sessions with needle and moxibustion on BL65 and SI1. Of these, 4 (4.3%) had fetal upheaval and natural childbirth with breech presentation.

Overall, 58 over 93 women (62.4%) with breech presentation had foetal upheaval and all had natural childbirth with uncomplicated vaginal delivery. In our study successful foetal upheaval was related to compliance to home treatment (p<0.005) and did not depend on the mother's age, nationality, and parity (data not shown).

Among the 60 primiparae (aged 18 to 45 years, mean age 32 years), 38 (63.3%) had cephalic presentation at delivery and natural childbirth. Among the 33 multiparous women (aged 27 to 40 years, mean age 35 years), 20 (60.7%) had cephalic presentation at delivery and natural childbirth.

Table 1 shows the occurrence of foetal upheaval divided by the week of pregnancy in which treatment was started. There was no significant difference in foetal upheaval in the primiparae, but the multiparae showed significantly more foetal upheaval in the later weeks (P<0.005).

In the first days of treatment we observed 2 pregnant women with increased uterine contractile activity (but not powerful enough to discontinue treatment) and one woman with premature rupture of membranes, which led to emergency caesarean section. No other side effects were noted.

## 4. Discussion

Our case series showed that in women with breech presentation self-administration of moxibustion on BL67 in a position with knees and hips flexed and with an anterior tilt of the pelvis, followed by acupuncture in case of failure of moxibustion, resulted in cephalic presentation and natural birth in 100% of the treated women. Other authors have reported a success rate in terms of in cephalic presentation in 55-70% of the women treated. We presume that this results from the combination of different factors. One factor could be the compliance in the self-administration of the moxibustion treatment, which was significantly related to successful foetal upheaval. Compliance has been shown to be extremely relevant in a negative study by Cardini et al. [13], where the possible combination of cultural factors together with a lack of trust in the treatment may have led to a high dropout from the trial. Since then many studies have been published on the effects of BL67 moxibustion for foetal reversion, and the procedure may have become more conventional and thus more easily accepted.

Another factor could be the position of the woman receiving the moxibustion treatment.

The school of obstetrics and gynecology in Classical Chinese Medicine recognizes in Chen Zi-Ming (1190-1270 AD, Song dynasty) his father, despite the fact that other famous previous authors (Dai Xia Yi, Zhang Zhong Jing, Chao Yuan Fang, and Sun Si Miao) have dedicated chapters of their books to gynecology and obstetrics. The work of Chen Zi-Ming gives greater importance to Qi (Yang) than Blood (Yin) because the former is of fundamental importance for the movement of the second. In the dynamics of birth the foetus represents the Yang and his mother the Yin. The foetus thus has the three Yang energy levels: Tai Yang for cephalic orientation, Shao Yang for the descent, and Yang Ming for the effort. The points to be used are the Jing-well acupoints. These are also the origins of the sinew channels, which carry Wei Qi, the most Yang energy of the body, responsible for the instinctive, automatic, and reflex movements. Tai Yang means Great Yang, the Yang which allows us to go where we want and which maintains our upright position. The cephalic position is like the foundation, the germ from which the journey of the unborn child will sprout. As these meridians regulate muscle movement, to treat them it is necessary that the muscles pertaining to them are not taut. Tai Yang develops along the back of the body, so for the opening of the acupoints it is necessary to adopt a position that relaxes the muscles in the paravertebral and thigh area, so that vertebral lordosis and kyphosis maintained by it disappear. Because in the antenatal period the mother is intermediary of the unborn child, not being able to directly treat the baby, we give the message and physical therapy to the mother, who sends it to the baby. Another consideration regarding the position of the pregnant woman can be made in terms of the discipline of Qi Gong. Qi Gong is a traditional Chinese technique which involves three pillars: posture, breathing, and intention. In the work of breathing is the idea that the forces of Heaven (related to Yang and light) are brought by respiration to Earth (linked to the Yin and darkness), which absorbs and transmits them to man. This dynamic reflects the alchemical relationship between Fire and Water. The meridians through which man receives the forces of Heaven mediated by Earth are those of Water, Yin for the mother (KI1) and Yang for the foetus (BL67). The posture with knees and hips flexed and pelvis tilted forward is typical of every style of Qi Gong that focuses on the Lower Dan Tian or the pelvis. The supine position stimulated the lung, the organ that manages the Wei Qi, while the mother practiced spontaneous breathing, the one automatic, instinctive, linked to Wei Qi. Thus position and respiration were designed to “let go,” so that what is spontaneous and natural could happen. The aspect of the intention was obtained suggesting that it was the father (the one who gives the rule to the son) to carry out the treatment of moxibustion.

It is known that the rate of spontaneous version is lower in nulliparous women and at higher gestational age. In our study we found that the results of the intervention in primiparous women are encouraging (63,3% had foetal upheaval and natural childbirth), while in multiparae they are not so different from what would be expected spontaneously (60.7%). We observed better results of treatment when it was administered in the more advanced weeks of gestation. The same results have been reported by Cardini et al. [13, 14]. This supports the impression of Cardini about an instability of the presentation at the 35th week of gestation in the Caucasian population. Albeit with numbers too small to be significant, we have seen encouraging results with delayed treatment (after 36th week) with acupuncture and moxibustion of BL65 and SI1 in the case of self-administered moxibustion of BL67 ineffective.

Our double-step strategy has further increased the foetal upheavals, limiting the need of the doctor. Another similar study with the use of acupuncture and moxibustion achieved global success of 53.6% [15], but the need for the presence of the doctor (in Italy acupuncture must be administered by a doctor) commits more resources. In our study the doctor intervened only in case of failure of the self-administered moxibustion. In the study only 12.9% (12/93) women made use of the provision of doctor.

In conclusion, the results obtained in this case series with self-administered moxibustion and rescue acupuncture can be considered promising in increasing the revolution of the foetus breech and in facilitating the reduction of deliveries by caesarean section, especially in primiparous. This approach also encourages education in self-care. Self-administered moxibustion is a simple, cost-effective technique that requires no medical intervention and should be offered to all women with a breech presentation because it is noninvasive and can be self-administered by the woman. We suggest starting treating the multiparae at the 34th or 35th week.

## Figures and Tables

**Table 1:** Occurrence of foetal upheaval divided by the week of pregnancy in which treatment was started.

Foetal upheaval	33^rd^ week n/total (%)	34^th^ week n/total (%)	35^th^ week n/total (%)	36^th^ week n/total (%)	p value
Primiparae	13/22 (59.1%)	17/27 (63.0%)	7/10 (70.0%)	1/1 (100%)	P = 0.7 (NS)
Multiparae	0/5 (0%)	11/18 (61.1%)	9/10 (90%)		P < 0.005

## Data Availability

The ultrasound data for diagnosis of foetal breech and the written informed consent forms used to support the findings of this study have not been made available because a flood destroyed the archives.
